# Dataset for the comparison of vacuum-treated and as-etched porous silicon samples in terms of the evolution of oxidation at low temperatures

**DOI:** 10.1016/j.dib.2020.105475

**Published:** 2020-04-08

**Authors:** Arturo Ramírez-Porras, Kevin Allen, Juan S. Pereira-Cubillo

**Affiliations:** a Centro de Investigación en Ciencia e Ingeniería de Materiales (CICIMA), Universidad de Costa Rica, San Pedro 11501, San José, Costa Rica; b Escuela de Física, Universidad de Costa Rica, San Pedro 11501, San José, Costa Rica

**Keywords:** Porous silicon, Oxidation, Infrared spectroscopy, Photoluminescence

## Abstract

The development of chemical sensors made from porous silicon is a task that has been addressed for several years. In order to have a reliable sensing material, stability must be guaranteed. Oxidation in silicon degrades the sensing capability. The data presented in this article provides some important insights concerning the treatment of samples that can improve the material stability against oxidation. For this purpose, Fourier Transformed Infrared (FTIR) measurements using an Attenuated Total Reflectance (ATR) additament were employed to extract information concerning oxidation on the samples submitted to different temperatures. Photoluminescent (PL) measurements were also performed on the samples in order to extract information on nanocrystals sizes and their relationship with oxidation.

Specifications table

**Table:** 

Subject	Materials Chemistry
Specific subject area	Semiconductor surfaces electrochemical treatment to produce nanocrystalline material for the development of new chemical sensors
Type of data	Table Graph
How data were acquired	• ATR data: FTIR spectroscopy using instrument with ATR additament. • Photoluminescence (PL) data: UV/VIS spectroscopy using a CCD-based spectrometer, a UV laser as a light source and fiber optics. • Data fittings: PeakFit software and already published models for fitting methodology.
Data format	Raw Analyzed
Parameters for data collection	All data were acquired in a closed laboratory room, with relative humidity around 40% and a temperature near 20 °C. ATR measurements required an additament with varying temperature capability. PL data were recorded using a 5 mW laser and optical density filters for attenuation. All data processes (plotting, fitting) were performed using desktop computers.
Description of data collection	ATR: Setting of working temperature by an external controller and wait a few minutes for stabilization; reflectance data acquisition from software-driven instrument; software data process to exchange reflectance to absorbance measurements. PL: Focusing attenuated laser light onto sample surface and registration of PL light by an optic probe connected to software-driven spectrometer.
Data source location	Institution: CICIMA, Universidad de Costa Rica City/Town/Region: San Pedro de Montes de Oca Country: Costa Rica
Data accessibility	Repository name: Mendeley Data identification number: [provide number] Direct URL to data: [e.g., https://www.data.edu.com]
Related research article	K. Allen, J.S. Pereira-Cubillo, A. Ramírez-Porras, Vacuum treatment do stabilize oxidation at low temperature region in porous silicon, https://doi.org/10.1016/j.apsusc.2019.144240

## Value of the Data

• Data provides valuable information concerning the comparison between porous silicon samples stored in high vacuum right after synthesis and non-treated samples (as-etched).
• Materials scientists and engineers developing chemical sensors can benefit from these data.
• These data are useful in the study of oxidation effects in the semiconductor, providing a better understanding of the material.

## 1 Data description

**Fig. 1:**
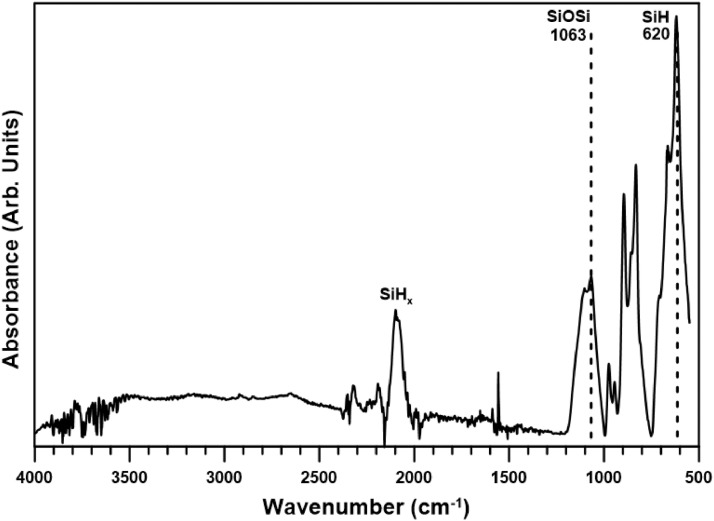
FTIR Absorbance spectrum for a pSi as-etched sample obtained at 21 °C. Important SiH bending and SiOSi asymmetric stretch modes are shown at 620 cm^−1^ and 1063 cm^−1^, respectively. SiH_x_ stretch modes are also marked near 2100 cm^−1^.

The main data files can be retrieved in the link provided in appendix A. FTIR-ATR Absorbance data for *as-etched* samples and *vacuum treated* samples (see details in the next section) for different temperatures from 21 °C to 202 °C are provided. Fig. 1 shows a plot for an as-etched sample at 21 °C. Some important features are also shown. The importance of the SiOSi and SiH band are fully discussed in [1].

Tables 1 and 2 show the temperatures, inverse temperature, amplitude of SiH peak, amplitude of SiOSi peak and SiOSi-to-SiH amplitude ratios for the As-etched sample (Table 1) and Vacuum treated sample (Table 2). The amplitudes can be extracted from the FTIR-ATR data mentioned above.

**Table 1:** Temperatures, inverse temperature, peak amplitude of modes and ratio of amplitudes for the As-etched sample.

Temp (°C)	Temp (K)	1000/T (K^−1^)	Amplitude SiH peak at 620 cm^−1^	Amplitude SiOSi peak at 1063 cm^−1^	SiOSi/SiH
21	294	3.4014	1.3080	0.5054	0.3864
41	314	3.1847	1.3090	0.5074	0.3876
61	334	2.9940	1.2465	0.5130	0.4116
81	354	2.8249	1.2249	0.5301	0.4328
101	374	2.6738	1.0887	0.5639	0.5180
121	394	2.5381	0.9745	0.6126	0.6286
141	414	2.4155	0.8080	0.6734	0.8334
161	434	2.3041	0.6496	0.7201	1.1085
181	454	2.2026	0.5501	0.7984	1.4514
201	474	2.1097	0.4274	0.8133	1.9029

**Table 2:** Temperatures, inverse temperature, peak amplitude of modes and ratio of amplitudes for the Vacuum treated sample.

Temp (°C)	Temp (K)	1000/T (K^−1^)	Amplitude SiH peak at 620 cm^−1^	Amplitude SiOSi peak at 1063 cm^−1^	SiOSi/SiH
22	295	3.39	1.2448	0.6291	0.5054
42	315	3.17	1.2474	0.6331	0.5075
62	335	2.99	1.2484	0.6368	0.5101
82	355	2.82	1.2441	0.6395	0.5140
102	375	2.67	1.2020	0.6514	0.5419
122	395	2.53	1.1159	0.6729	0.6030
142	415	2.41	0.9285	0.7161	0.7712
162	435	2.30	0.6607	0.7387	1.1181
182	455	2.20	0.4510	0.7477	1.6579
202	475	2.11	0.3199	0.7511	2.3479

**Fig. 2:**
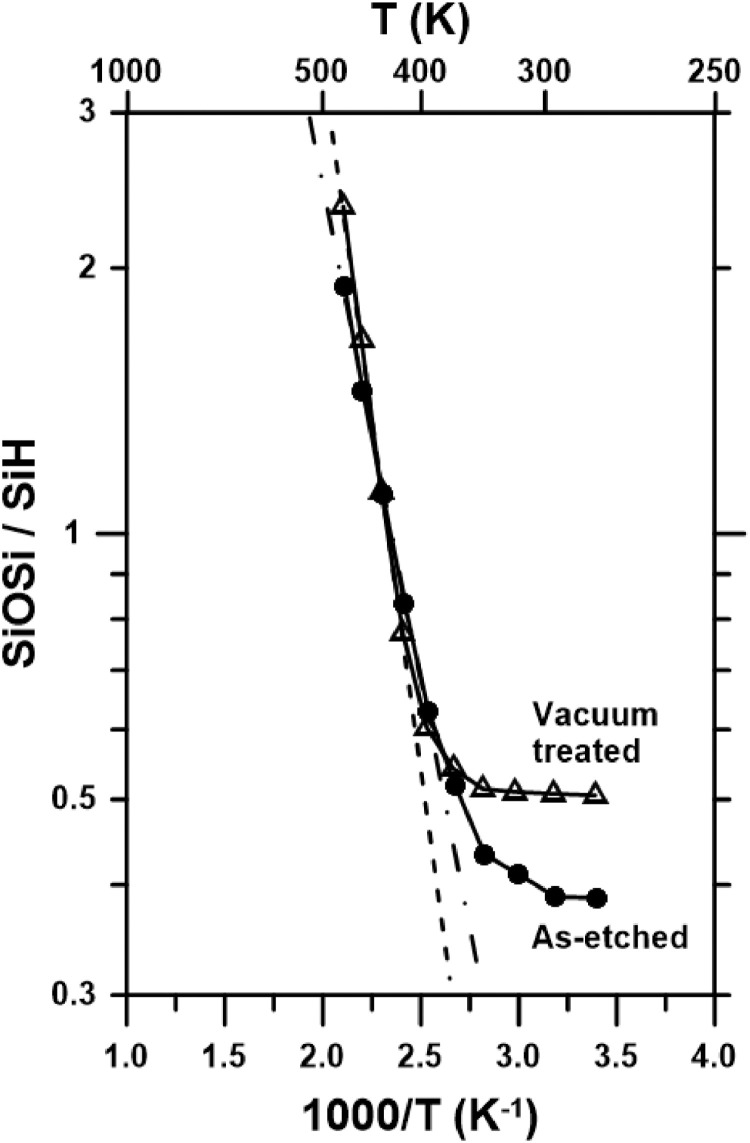
Peak amplitude relations between SiOSi and SiH bands as a function of the inverse of temperature for the As-etched sample and the Vacuum treated sample. The dotted lines correspond to Arrhenius fits for temperatures higher than around 400 K.

Fig. 2 plots the SiOSi/SiH ratio against the inverse temperature for both kinds of samples. The behaviors are different and are explained in [1].

**Fig. 3:**
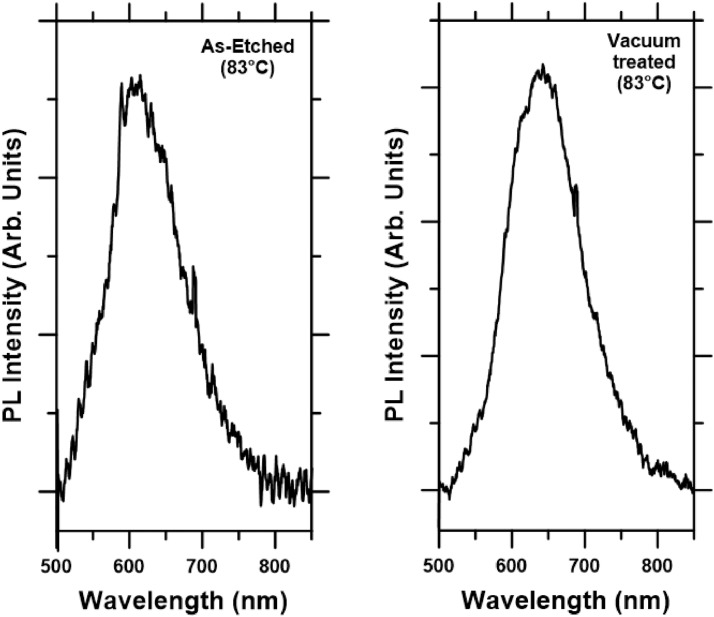
PL spectra of As-etched (left) and Vacuum treated (right) samples in the visible region.

Data files also contain PL measurements of As-etched and Vacuum treated samples for three temperatures: 21 °C, 83 °C and 130 °C. As an example, Fig. 3 shows PL plots in the visible region of the As-etched and the Vacuum treated samples after being exposed to a temperature of 83 °C. From these data, contributions from quantum dots (QD), quantum wires (QW) and their surface localized states (Loc. States) can be extracted according to the methodology explained in [2].

## 2 Experimental design, materials, and methods

PSi samples were obtained from Boron-doped crystalline Silicon, (100) surface indices, and 20–50 Ohm-cm resistivity using an electrochemical etching cell filled with an ethanoic HF solution at 12.5% of acid concentration. A constant current density of 54 mA.cm^−2^ was used in the process for a 6 min etching time. The samples were subsequently classified into two groups: the ones with no further treatment after production, were named *as-etched*, whereas those who were exposed to a high vacuum system using turbomolecular pumps (base pressure below 1 × 10^−6^ Torr for at least 24 h) were named *vacuum treated*. For both kinds of samples, the following surface characterizations were performed: Fourier Transformed Infrared spectroscopy with Attenuated Total Reflectance additament (FTIR-ATR, Frontier model of PerkinElmer employing a diamond crystal in contact with the samples, in the energy range from 550 to 4000 cm^−1^. Infrared measurements with temperature variation could be obtained using a Pike GladiATR accessory connected to the Frontier FTIR. The temperature ranged from 21 °C to 201 °C. In these circumstances, the whole oxidation process took less than one hour. Photoluminescence (PL) data were recorded using a two-channel Ocean Optics (model SD-2000) spectrophotometer with a 447 nm laser as a light source connected with a fiber-optic probe. PL analysis to extract nanocrystalline parameters was performed as indicated in [2].

## Appendix Supplementary materials

Image, application 1
